# Efficient Recycling of Gold and Copper from Electronic Waste by Selective Precipitation

**DOI:** 10.1002/anie.202308356

**Published:** 2023-08-29

**Authors:** Abhijit Nag, Mukesh K. Singh, Carole A. Morrison, Jason B. Love

**Affiliations:** ^1^ EaStCHEM School of Chemistry University of Edinburgh EH9 3FJ Edinburgh UK

**Keywords:** Au Precipitation, Electronic Wastes, Noble Metals, Recycling, Sustainable Chemistry

## Abstract

The recycling of metals from electronic waste (e‐waste) using efficient, selective, and sustainable processes is integral to circular economy and net‐zero aspirations. Herein, we report a new method for the selective precipitation of metals such as gold and copper that offsets the use of organic solvents that are traditionally employed in solvent extraction processes. We show that gold can be selectively precipitated from a mixture of metals in hydrochloric acid solution using triphenylphosphine oxide (TPPO), as the complex [(TPPO)_4_(H_5_O_2_)][AuCl_4_]. By tuning the acid concentration, controlled precipitation of gold, zinc and iron can be achieved. We also show that copper can be selectively precipitated using 2,3‐pyrazinedicarboxylic acid (2,3‐PDCA), as the complex [Cu(2,3‐PDCA‐H)_2_]_n_ ⋅ 2n(H_2_O). The combination of these two precipitation methods resulted in the recovery of 99.5 % of the Au and 98.5 % of the Cu present in the connector pins of an end‐of‐life computer processing unit. The selectivity of these precipitation processes, combined with their straightforward operation and the ability to recycle and reuse the precipitants, suggests potential industrial uses in the purification of gold and copper from e‐waste.

## Graphical abstract

Efficient and selective recovery of gold and copper is achieved using triphenylphosphine oxide and 2,3‐pyrazinedicarboxylic acid, respectively from 2 M HCl e‐waste leach solutions. The selectivity of these precipitation methods, together with their ease of operation and capacity to recycle and reuse the precipitants, offers potential applications in the purification of Au and Cu from various metal sources.

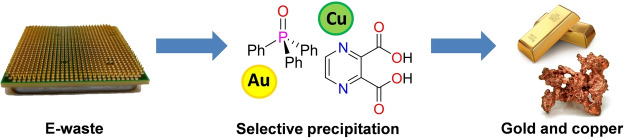

The recycling of metals from electronic waste (e‐waste), end‐of‐life industrial or automotive catalysts, fuel cells, and batteries presents significant economic, environmental, and net‐zero opportunities.[^1^, ^2^, ^3^] Currently, 93.5 million tons of e‐waste are generated globally each year, with waste printed circuit boards (PCBs) accounting for approximately 50 million tons.^[4]^ E‐waste is a concentrated source of base metals such as iron, nickel, copper, and zinc as well as many high‐value noble metals that include silver, gold, platinum, and palladium.^[5]^ In particular, the Cu content of waste PCBs is 20 % by weight, while the Au content of waste mobile phones is up to 1200 g/t; these concentrations are far higher than those found in natural Cu and Au minerals.^[6]^ As a result, the overall economic benefits of a single recycling process for metal wastes will be greatly enhanced if noble metals and other valuable metals like Cu, Zn, and Fe can be selectively recovered and returned to active use.

While the sustainable recycling of these materials is complex and often uses environmentally unsustainable methods, the development of new hydrometallurgical processes for e‐waste recycling has received recent attention because of the potential reduction in environmental impact, suitability for small scale applications, and low capital cost.[^7^, ^8^, ^9^, ^10^, ^11^, ^12^, ^13^, ^14^] In this context, highly selective, reusable precipitation and adsorption methods are becoming popular as they offer advantages over traditional, single‐use precipitants and avoid the use of organic solvents required in solvent extraction technologies.[^15^, ^16^, ^17^, ^18^, ^19^, ^20^] These strategies rely on molecular and supramolecular chemical recognition processes and the propensity to form designed structural motifs. For example, pre‐organized cyclodextrin, cucurbituril, and macrocyclic tetralactam receptors were shown to host selectively Au or Pt anions within spontaneously assembled superstructures that precipitate from aqueous acidic solutions.[^21^, ^22^, ^23^, ^24^] Simple biomolecules such as niacin forms an extended supramolecular network with Au anions under acidic conditions that allows its separation from base, alkali, and alkaline‐earth metals.^[25]^ Similarly, Au precipitation as extended networks resulting from supramolecular interactions between acyclic durene diamides and HAuCl_4_ was seen, albeit with no selectivity reported.^[26]^ More recently, the selective precipitation of Au from an aqueous mixed‐metal solution obtained from the aqua regia leaching of e‐waste was demonstrated using a simple tertiary diamide.^[27]^ In this example, protonating the diamide forms a cationic receptor which assembles into supramolecular chains in the solid state to form cavities ideal for the encapsulation of the square‐planar anion AuCl_4_^−^. Furthermore, the uptake of different metals such as Fe, Ga, Zn, and Pt was tuned by varying the HCl concentration, making this system highly versatile and applicable to a range of metal separation scenarios.

Herein, we report the selective precipitation of Au and Cu from metal mixtures in aqueous acid using very simple, readily obtainable and recyclable precipitating reagents. Selective uptake of Au by triphenylphosphine oxide (TPPO) is seen, forming the ion pair [(TPPO)_4_(H_5_O_2_)][AuCl_4_] **1** as characterised by single crystal X‐ray diffraction (SC‐XRD), UV/Vis spectrophotometry, electrospray ionisation mass spectrometry (ESI‐MS) and nuclear magnetic resonance (NMR) spectroscopy. Furthermore, the selective uptake of Cu by 2,3‐pyrazinedicarboxylic acid (2,3‐PDCA) as the polymeric complex [Cu(2,3‐PDCA‐H)_2_]_n_ ⋅ 2n(H_2_O) **2** is established from a mixture of metals in HCl. By combining these two methods, a process is developed through which 99.5 % Au and 98.5 % Cu are selectively recycled in high purity from the connector pins of an end‐of‐life central processing unit (CPU).

The addition of solid TPPO (0.28 mmol) to a stirred solution of HAuCl_4_ (5 mL, 10 mM) in 2 M HCl at room temperature (RT) results in the immediate formation of a yellow precipitate (Figure 1). Analysing the solution before and after the addition of TPPO by ICP‐OES showed that 99.5 % of Au is precipitated, with this efficiency of precipitation retained in water and 2, 4, and 6 M HCl (Figure S1). The concentration threshold for gold precipitation was explored by varying HAuCl_4_ concentration (0.01 to 50.0 ppm) with constant TPPO and showed a drop off in gold uptake from 96.5 % (by ICP‐MS) at 5.0 ppm HAuCl_4_ to 83.3 % at 1.0 ppm, with 24.7 % uptake seen at 0.1 ppm (Table S1, Figure S2). It is therefore evident that while this method is effective for recovering low concentrations of gold from solutions relevant to e‐waste recycling it is not usable for trace gold uptake. The gold‐containing precipitate was dissolved in acetonitrile and reduced using NaBH_4_ to release metallic Au from the complex and recycle the TPPO which is retained in the organic solvent, as revealed by the resonance at 28.5 ppm in the ^31^P{^1^H} NMR spectrum (Figure 1 and S3).

**Figure 1:**
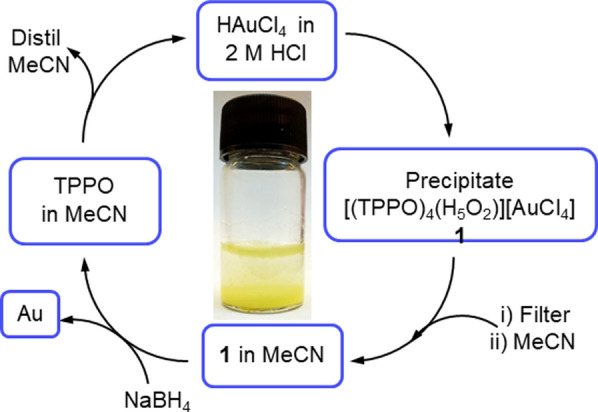
Schematic representation of the recovery of Au by precipitation using TPPO (Ph_3_P=O) in 2 M HCl and reduction of Au(III) complex using NaBH_4_ to form metallic Au and recycle TPPO.

The Au‐containing precipitate was crystallised by vapour diffusion of diethyl ether into a dichloromethane solution and the single‐crystal X‐ray structure determined (Figures 2 and S4, Table S2) as [(TPPO)_4_(H_5_O_2_)][AuCl_4_], **1**.^[42]^ The expanded structure (Figure 2A) shows that the TPPO molecules pack to generate two types of pocket, one containing the anion AuCl_4_^−^ in an aryl pocket and the other the (H_5_O_2_)^+^ cation, bound through classical hydrogen bonding with the phosphine oxide (O3−(H)−O3′=2.424(2) Å, O1−(H)−O3=2.535(2) Å and O2−(H)−O3=2.584(2) Å, Figure 2C). The powder X‐ray diffraction pattern of the Au‐containing precipitate was recorded and matches that simulated from the single‐crystal X‐ray data, confirming that the bulk and single‐crystal materials are structurally cohesive (Figure S5). The UV/Vis spectrum of **1** in acetonitrile displays absorptions at 270 and 320 nm which are attributed to those for TPPO and [AuCl_4_]^−^, respectively (Figure S6A). The identity of **1** is further supported by a resonance at 34.86 ppm in the ^31^P{^1^H} NMR spectrum which is due to protonated TPPO (Figure S6B).^[28]^ Similarly, the positive and negative‐ion ESI‐MS spectra (acetonitrile) of **1** show the presence of ions consistent with TPPO and AuCl_4_^−^ (Figure S7A and B).

**Figure 2:**
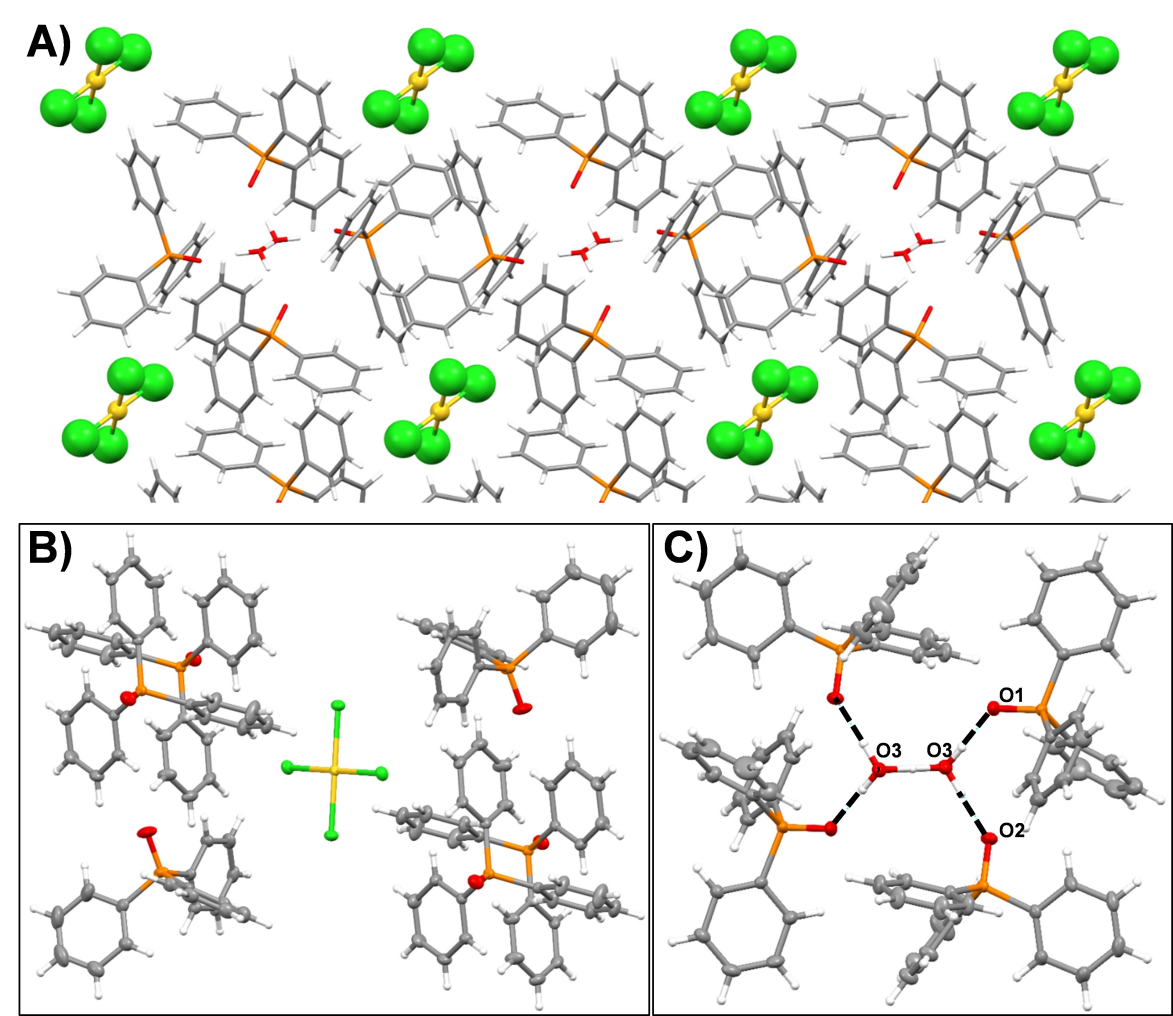
The X‐ray crystal structure of **1**, colour codes: C, grey; Au, yellow; P, orange; O, red; Cl, green; and H, white. A) Expanded view showing two pockets in which H_5_O_2_^+^ and AuCl_4_^−^ reside. B) Aryl pocket hosting AuCl_4_^−^ anion. C) Classical hydrogen‐bonding interactions between H_5_O_2_^+^ and TPPO.

The selectivity of TPPO for Au was evaluated from equimolar mixtures of HAuCl_4_, ZnCl_2_, FeCl_3_, PdCl_2_, NiCl_2_, H_2_PtCl_6_ and CuCl_2_ (10 mM each) in 2 M and 6 M HCl. Stirring the mixed‐metal solution with TPPO (0.36 mmol) in 2 M HCl for 1 h resulted in the precipitation of Au (99.5 %) and Zn (94.5 %) only (Figure S8A), which upon washing with 6 M HCl resulted in the release of Zn into solution and the retention of Au in the precipitate (Figure S8B). In contrast, at 6 M HCl precipitation of Au (99.5 %) and Fe (70.5 %) occurred (Figure S8C), which upon washing with water released the Fe back into solution while retaining the Au in the precipitate (Figure S8D). The uptake of Fe by TPPO at 6 M HCl but not at 2 M HCl is likely due to the increased amount of FeCl_4_^−^ present on increasing the HCl concentration.^[27]^ Previous studies on the precipitation of ZnCl_2_ by TPPO from polar organic solvents show that complexation of ZnCl_2_ by TPPO occurred.^[29]^ Crystals from the precipitation of Fe by TPPO in 6 M HCl were also obtained in this work, and the unit cell data match those of the previously reported complex [FeCl_2_(TPPO)_4_][FeCl_4_].^[30]^ The identity of this complex is further supported by ESI‐MS of an acetonitrile solution of the Fe‐containing precipitate which shows the presence of [FeCl_2_(TPPO)_2_]^+^ in positive ion mode and [FeCl_4_]^−^ in negative ion mode (Figure S9A and B). As such, through varying the acid concentration and the stripping step, the selective recovery of Au, Zn and Fe from a mixed‐metal solution is achieved.

The separation and purification of Cu from e‐waste is highly important, as it typically is the major component of any metallic component, accounting, for example, for 23.4 wt % of the metal present in PCBs.^[8]^ The precipitation of Cu(II) salts from industrial effluents through the formation of insoluble sulfides and other complexes is long recognised,[^31^, ^32^, ^33^, ^34^] and is complementary to the use of pyridine carboxylates[^35^, ^36^, ^37^] or phenolic oxime reagents[^38^, ^39^, ^40^] in the selective recovery of Cu by solvent extraction. These studies suggest that selective precipitation of Cu from acidic metal solutions may be achieved using 2,3‐pyrazinedicarboxylic acid (2,3‐PDCA, Figure 3). It is known that reactions between Cu(II) salts and 2,3‐PDCA result in the formation of polymeric materials such as [Cu(2,3‐PDCA‐H)_2_]_n_ ⋅ 2n(H_2_O) and [Cu(2,3‐PDCA)(OH_2_)_2_]_n_ which exhibit both coordination and supramolecular interactions in their structures.^[41]^

**Figure 3:**
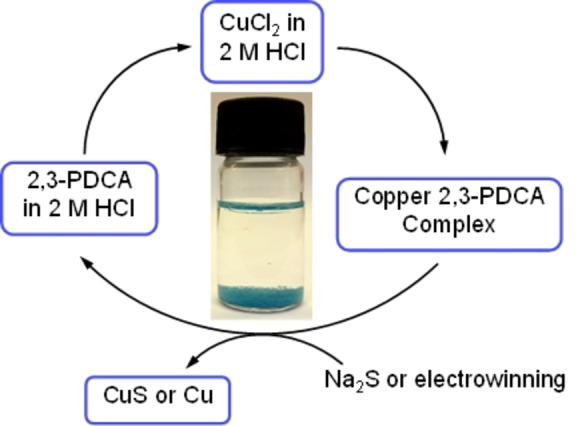
Schematic representation of precipitation of Cu using 2,3‐PDCA from 2 M HCl.

The addition of solutions of 2,3‐PDCA (100 mM, in 2 M or 6 M HCl) to solutions of CuCl_2_ (10 mM, in 2 M or 6 M HCl) results in the complete precipitation of Cu as [Cu(2,3‐PDCA‐H)_2_]_n_ ⋅ 2n(H_2_O) **2** from 2 M HCl but no precipitation from 6 M HCl (Figure S10). Similarly, complete precipitation of Cu is also observed using Na_2_(2,3‐PDCA) from 2 M HCl. Treatment of **2** with Na_2_S results in the formation of CuS and the release of Na_2_(2,3‐PDCA) into the aqueous solution, as revealed by ^13^C and ^1^H NMR spectroscopy (Figures S11A and S11B). Alternatively, Cu deposits in its metallic form by electrochemical reduction of a solution of **2** in 6 M HCl (Figure S12A and S12B). Analysis of crystals of **2** by X‐ray crystallography reveals its identity to be [Cu(2,3‐PDCA‐H)_2_]_n_ ⋅ 2n(H_2_O) through matching the unit cell parameters to the previously reported structure (see Figure S13 for the extended structure).^[41]^

The selectivity of Cu precipitation by 2,3‐PDCA (100 mM, in 2 M HCl) was evaluated using an equimolar mixture of HAuCl_4_, ZnCl_2_, FeCl_3_, PdCl_2_, NiCl_2_, H_2_PtCl_6_ and CuCl_2_ (10 mM each) in 2 M HCl. Significantly, this results in the selective precipitation of Cu (99.0 %) with only minimal uptake of Pd (5.8 %), Pt (2.5 %) and Au (2.4 %) (Figure S14). An advantage of using the 2,3‐PDCA ligand is that an extended supramolecular structure is formed which, along with Irving–Williams stability considerations, likely favours selective copper uptake.

Combining the two approaches described above for acidic mixed‐metal solutions allows a protocol for the recovery of Au and Cu from e‐waste to be devised (Figure S15). To test this, the recovery of Au and Cu from the connector pins of a waste CPU, comprising Ni, Cu and Au was undertaken.[^2^, ^26^] The connector pins (ca. 88 mg) were dissolved in 2 M HCl/H_2_O_2_ (Figure 4A and B), resulting in a solution of Ni (658 ppm), Cu (11190 ppm), and Au (101 ppm). The addition of TPPO (0.18 mmol) to this solution results in 99.5 % precipitation of Au in 2 h (Figures 4C). This was recovered by filtration, dissolved in acetonitrile (Figure 4D) which can be reduced using NaBH_4_ to create metallic Au and regenerate the TPPO. The remaining filtrate was treated with 2,3‐PDCA (200 mM) to precipitate 98.5 % of the Cu, with minimal precipitation of Ni (1.0 %) (Figure 4E and 4F). Hence, it is clear that TPPO and 2,3‐PDCA are highly selective and efficient for the recovery of Au and Cu, respectively, from this particular e‐waste source. Using this method, about 99.5 % pure Au and 99.0 % pure Cu from e‐waste can be achieved.

**Figure 4:**
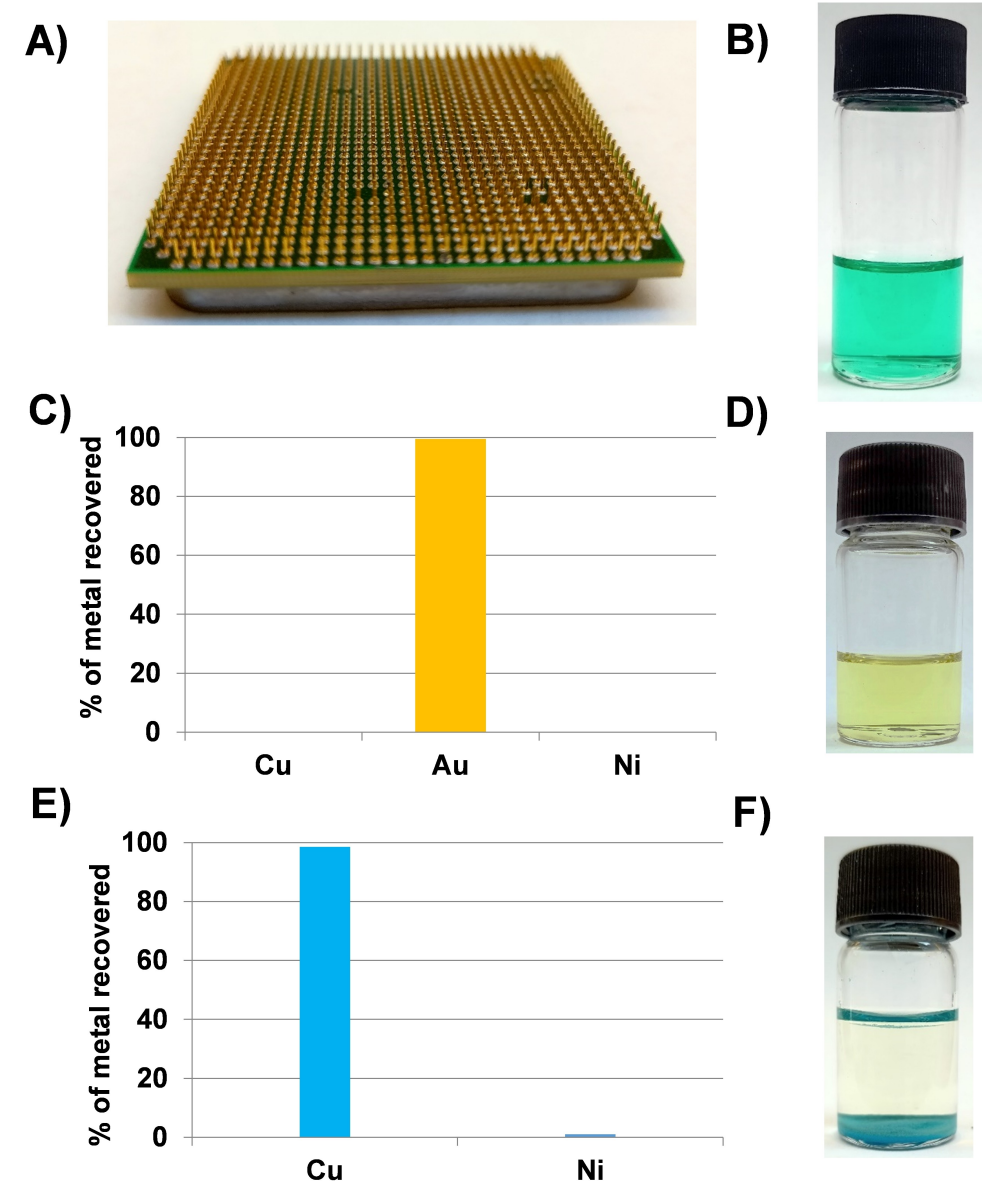
Separation of Au, Cu, and Ni from the connector pins of an end‐of‐life CPU. A) Image of the CPU connector pins. B) Solution obtained after dissolution into 2 M HCl/H_2_O_2_. C) Percentage of Au recovery from the solution in B on addition of TPPO. D) Resulting solution obtained upon dissolving the Au precipitate in acetonitrile. E) Percentage of Cu recovery on addition of 2,3‐PDCA. F) Image of the resulting Cu‐2,3‐PDCA precipitate, with the Ni ions remaining in solution.

In summary, the separation of Au from mixed‐metal HCl solutions was achieved by selective precipitation on addition of TPPO, to generate the complex [(TPPO)_4_(H_5_O_2_)[AuCl_4_], **1**. By adjusting the concentration of HCl other metal ions such as Zn^2+^ and Fe^3+^ can be separated from co‐existing metal ions found in leach solutions of e‐waste, such as Cu^2+^, Ni^2+^, Pt^4+^, and Pd^2+^. We have also discovered that 2,3‐PDCA can be used to selectively precipitate Cu from a mixed‐metal solution in 2 M HCl. This has allowed a process to be developed for the selective recovery of Au and Cu from e‐waste via precipitation, which negates the need for classical solvent extraction techniques and their reliance on environmentally hazardous organic solvents. Importantly, and unlike classical sulfidic precipitation methods, the reagents used in this work are straightforwardly recycled and reusable. Furthermore, these precipitation protocols may potentially be exploited more widely to recover Au and Cu from ores, spent catalysts, electronic waste, and nano‐waste in a sustainable manner.

## Conflict of interest

The authors declare no conflict of interest.

## Supporting information

As a service to our authors and readers, this journal provides supporting information supplied by the authors. Such materials are peer reviewed and may be re‐organized for online delivery, but are not copy‐edited or typeset. Technical support issues arising from supporting information (other than missing files) should be addressed to the authors.

Supporting Information

Supporting Information

## Data Availability

The data that support the findings of this study are available from the corresponding author upon reasonable request.

## References

[1] M. D. Rao , K. K. Singh , C. A. Morrison , J. B. Love , RSC Adv. 2020, 10, 4300–4309. 35495234 10.1039/c9ra07607g PMC9049023

[2] C. Yue , H. Sun , W.-J. Liu , B. Guan , X. Deng , X. Zhang , P. Yang , Angew. Chem. Int. Ed. 2017, 56, 9331–9335. 10.1002/anie.201703412 28613435

[3] A. Zupanc , J. Install , M. Jereb , T. Repo , Angew. Chem. Int. Ed. 2023, 62, e202214453. 10.1002/anie.202214453 PMC10107291 36409274

[4] L. Zhang , Z. Xu , J. Cleaner Prod. 2016, 127, 19–36.

[5] B. Deng , D. X. Luong , Z. Wang , C. Kittrell , E. A. McHugh , J. M. Tour , Nat. Commun. 2021, 12, 5794. 34608143 10.1038/s41467-021-26038-9 PMC8490403

[6] R. H. Estrada-Ruiz , R. Flores-Campos , H. A. Gámez-Altamirano , E. J. Velarde-Sánchez , J. Hazard. Mater. 2016, 311, 91–99. 26963241 10.1016/j.jhazmat.2016.02.061

[7] A. Baksi , M. Gandi , S. Chaudhari , S. Bag , S. S. Gupta , T. Pradeep , Angew. Chem. Int. Ed. 2016, 55, 7777–7781. 10.1002/anie.201510122 27119514

[8] M. Räisänen , E. Heliövaara , F. a. Al-Qaisi , M. Muuronen , A. Eronen , H. Liljeqvist , M. Nieger , M. Kemell , K. Moslova , J. Hämäläinen , K. Lagerblom , T. Repo , Angew. Chem. Int. Ed. 2018, 57, 17104–17109. 10.1002/anie.201810447 30370970

[9] A. Zupanc , E. Heliövaara , K. Moslova , A. Eronen , M. Kemell , Č. Podlipnik , M. Jereb , T. Repo , Angew. Chem. Int. Ed. 2022, 61, e202117587. 10.1002/anie.202117587 PMC9305299 35106899

[10] W. Lin , R.-W. Zhang , S.-S. Jang , C.-P. Wong , J.-I. Hong , Angew. Chem. Int. Ed. 2010, 49, 7929–7932. 10.1002/anie.201001244 20853302

[11] F. Forte , S. Riaño , K. Binnemans , Chem. Commun. 2020, 56, 8230–8232. 10.1039/d0cc02298e 32555853

[12] A. Serpe , L. Marchiò , F. Artizzu , M. L. Mercuri , P. Deplano , Eur. J. Chem. 2013, 19, 10111–10114. 10.1002/chem.201300940 23788281

[13] Y. Chen , M. Xu , J. Wen , Y. Wan , Q. Zhao , X. Cao , Y. Ding , Z. L. Wang , H. Li , Z. Bian , Nat. Sustainability 2021, 4, 618–626.

[14] A. Nag , C. A. Morrison , J. B. Love , ChemSusChem 2022, 15, e202201285. 35929761 10.1002/cssc.202201285 PMC9804267

[15] Z. Zhou , W. Zhong , K. Cui , Z. Zhuang , L. Li , L. Li , J. Bi , Y. Yu , Chem. Commun. 2018, 54, 9977–9980. 10.1039/c8cc05369c 30123906

[16] M. Mon , J. Ferrando-Soria , T. Grancha , F. R. Fortea-Pérez , J. Gascon , A. Leyva-Pérez , D. Armentano , E. Pardo , J. Am. Chem. Soc. 2016, 138, 7864–7867. 27295383 10.1021/jacs.6b04635

[17] D. T. Sun , N. Gasilova , S. Yang , E. Oveisi , W. L. Queen , J. Am. Chem. Soc. 2018, 140, 16697–16703. 30395464 10.1021/jacs.8b09555

[18] Y. Hong , D. Thirion , S. Subramanian , M. Yoo , H. Choi , H. Y. Kim , J. F. Stoddart , C. T. Yavuz , Proc. Natl. Acad. Sci. USA 2020, 117, 16174–16180. 32571947 10.1073/pnas.2000606117 PMC7368251

[19] T. S. Nguyen , Y. Hong , N. A. Dogan , C. T. Yavuz , Chem. Mater. 2020, 32, 5343–5349.

[20] F. Li , J. Zhu , P. Sun , M. Zhang , Z. Li , D. Xu , X. Gong , X. Zou , A. K. Geim , Y. Su , H.-M. Cheng , Nat. Commun. 2022, 13, 4472. 35918342 10.1038/s41467-022-32204-4 PMC9345893

[21] H. Wu , L. O. Jones , Y. Wang , D. Shen , Z. Liu , L. Zhang , K. Cai , Y. Jiao , C. L. Stern , G. C. Schatz , J. F. Stoddart , ACS Appl. Mater. Interfaces 2020, 12, 38768–38777. 32648728 10.1021/acsami.0c09673

[22] Z. Liu , M. Frasconi , J. Lei , Z. J. Brown , Z. Zhu , D. Cao , J. Iehl , G. Liu , A. C. Fahrenbach , Y. Y. Botros , O. K. Farha , J. T. Hupp , C. A. Mirkin , J. Fraser Stoddart , Nat. Commun. 2013, 4, 1855. 23673640 10.1038/ncomms2891 PMC3674257

[23] W. Liu , A. G. Oliver , B. D. Smith , J. Am. Chem. Soc. 2018, 140, 6810–6813. 29787255 10.1021/jacs.8b04155

[24] H. Wu , Y. Wang , L. O. Jones , W. Liu , L. Zhang , B. Song , X.-Y. Chen , C. L. Stern , G. C. Schatz , J. F. Stoddart , Angew. Chem. Int. Ed. 2021, 60, 17587–17594. 10.1002/anie.202104646 34031957

[25] A. Nag , M. R. Islam , T. Pradeep , ACS Sustainable Chem. Eng. 2021, 9, 2129–2135.

[26] C. C. Shaffer , W. Liu , A. G. Oliver , B. D. Smith , Eur. J. Chem. 2021, 27, 751–757. 10.1002/chem.202003680 32853413

[27] L. M. M. Kinsman , B. T. Ngwenya , C. A. Morrison , J. B. Love , Nat. Commun. 2021, 12, 6258. 34716348 10.1038/s41467-021-26563-7 PMC8556376

[28] T. Krachko , V. Lyaskovskyy , M. Lutz , K. Lammertsma , J. C. Slootweg , Z. Anorg. Allg. Chem. 2017, 643, 916–921.

[29] D. C. Batesky , M. J. Aufogel , D. J. Weix , J. Org. Chem. 2017, 82, 9931–9936. 28956444 10.1021/acs.joc.7b00459 PMC5634519

[30] V. Jorík , I. Ondrejkovicová , R. B. von Dreele , H. Ehrenberg , Cryst. Res. Technol. 2003, 38, 174–181.

[31] F. Fu , Q. Wang , J. Environ. Manage. 2011, 92, 407–418. 21138785 10.1016/j.jenvman.2010.11.011

[32] D.-H. Kim , M.-C. Shin , H.-D. Choi , C.-I. Seo , K. Baek , Desalination 2008, 223, 283–289.

[33] F. Akbal , S. Camcı , Desalination 2011, 269, 214–222.

[34] S. Petrov , V. Nenov , Desalination 2004, 162, 201–209.

[35] A. Wojciechowska , K. Wieszczycka , I. Wojciechowska , Sep. Purif. Technol. 2017, 185, 103–111.

[36] J. Szymanowski , G. Kyuchoukov , Can. Metall. Q. 2002, 41, 399–408.

[37] R. F. Dalton , A. Burgess , in Process Metallurgy, Vol. 7 (Ed.: T. Sekine ), Elsevier, 1992, pp. 1145–1150.

[38] K. Wieszczycka , M. Krupa , A. Olszanowski , Sep. Purif. Technol. 2012, 47, 1278–1284.

[39] K. Klonowska-Wieszczycka , A. Olszanowski , A. Parus , B. Zydorczak , Solvent Extr. Ion Exch. 2009, 27, 50–62.

[40] K. Wieszczycka , M. Kaczerewska , M. Krupa , A. Parus , A. Olszanowski , Sep. Purif. Technol. 2012, 95, 157–164.

[41] L. Mao , S. J. Rettig , R. C. Thompson , J. Trotter , S. Xia , Can. J. Chem. 1996, 74, 433–444.

[42] Deposition number 2269430 contains the supplementary crystallographic data for this paper. These data are provided free of charge by the joint Cambridge Crystallographic Data Centre and Fachinformationszentrum Karlsruhe Access Structures service.

