# Supplementary material for: Efficient Recycling of Gold and Copper from Electronic Waste by Selective Precipitation
Source: Angew Chem Int Ed Engl. 2023 Aug 29;62(40):e202308356. doi: 10.1002/anie.202308356 (PMC10952234; doi:10.1002/anie.202308356)
Supplement: Supplementary file 2 — Supporting Information [file ANIE-62-0-s002.pdf]

Angewandte Chemie
Eine Zeitschrift der Gesellschaft Deutscher Chemiker

Supporting Information

Efficient Recycling of Gold and Copper from Electronic Waste by Selective Precipitation

*A. Nag, M. K. Singh, C. A. Morrison, J. B. Love**

Supporting Information
©Wiley-VCH 2016
69451 Weinheim, Germany

Efficient Recycling of Gold and Copper from Electronic Waste by Selective Precipitation

Abhijit Nag, Mukesh K. Singh, Carole A. Morrison, and Jason B. Love*[a]

Abstract: The recycling of metals from electronic waste (e-waste) using efficient, selective, and sustainable processes is integral to circular economy and net-zero aspirations. Herein, we report a new method for the selective precipitation of metals such as gold and copper that negates the use of organic solvents that are traditionally employed in solvent extraction processes. We show that gold can be selectively precipitated from a mixture of metals in hydrochloric acid solution using triphenylphosphine oxide (TPPO), as the complex $[(TPPO)_4(H_5O_2)][AuCl_4]$. By tuning the acid concentration, selective precipitation control of gold, zinc and iron can be achieved. We also show that copper can be selectively precipitated 2,3-pyrazinedicarboxylic acid (2,3-PDCA), as the complex $[Cu(2,3\text{-PDCA-H})_2]_n$•$2n(H_2O)$. The combination of these two precipitation methods resulted in the recovery of 99.5% of the gold and 98.5% of the copper present in the connector pins on an end-of-life computer processing unit. The selectivity of these precipitation processes, combined with their straightforward operation and the ability to recycle and reuse the precipitants, suggests potential industrial uses in the purification of gold and copper from e-waste.

DOI: 10.1002/anie.2016XXXXX

[a] EaStCHEM School of Chemistry, University of Edinburgh,
Edinburgh, EH9 3FJ, UK.
E-mail: jason.love@ed.ac.uk

Supporting information for this article is given via a link at the end of the document

Materials and Methods

All reagents and solvents were used as obtained from Sigma-Aldrich, Fisher Scientific UK, Alfa Aesar, Acros Organics or VWR International. Deionised water was taken from a MilliQ purification system.

Crystallization of $[(TPPO)_4(H_5O_2)][AuCl_4]$ 1

150 mg of precipitate of the complex was dissolved in 1 mL dichloromethane and the sample vapour diffused using diethyl ether. After 3 days, yellow crystals of **1** suitable for single-crystal X-ray crystallographic analysis were obtained.

Crystallization of copper-2,3-PDCA complex 2

Briefly, 1 mL of $CuCl_2$ (10 mM) in 2 M HCl and 2 mL of 2,3-PDCA (100 mM) were mixed. The solution was kept at room temperature and, after 15 days, blue crystals of **2** were obtained. The crystal structure of this complex has been reported previously.[1]

Inductively Coupled Plasma Optical Emission Spectrometry (ICP-OES)

Quantitative metal analysis was carried out on a Perkin Elmer Optima 5300DC Inductively Coupled Plasma Optical Emission Spectrometer. For the measurements of metals in aqueous medium 2% HNO_3 was used; for the organic solvents 1-methoxy-2-propanol was used. For the aqueous sample measurements, the argon plasma conditions were 1550 W RF power, with gas flows of 15, 1.07, and 0.9 L min^{-1} for plasma, auxiliary, and nebuliser flows, respectively. For the organic sample measurements, the argon plasma conditions were 1550 W RF power, with gas flows of 17, 1.0, and 0.5 L min^{-1} for plasma, auxiliary, and nebuliser flow, respectively. Prior to sample data collection, the instrument was calibrated (R^2=0.9999) with metal standards, obtained from VWR International, Merck Millipore, or Sigma-Aldrich.

Inductively Coupled Plasma Mass Spectrometry (ICP-MS)

ICP-MS analysis at the ppb scale for gold solutions was carried out on an Agilent 7800 Single Quadrupole ICP-MS. Samples were prepared in 2% HNO_3 acid and were pumped by a peristaltic pump into a MicroMist nebulizer and a quartz Scott-type chamber at a rate of 0.3 reps. The plasma, auxiliary, and nebulizer gas flows were kept at 15, 1.07, and 0.91 L min^{-1}, respectively. The Argon plasma conditions were set at 1550W RF power.

Powder X-ray diffraction

Powder X-ray Diffraction (PXRD) data were collected using a Bruker D2 Phaser diffractometer in reflection geometry with Cu Kα radiation (λ = 1.541 Å). A LynxEye position sensitive detector was used to collect data for 20 min. Powder sample was spread on a zero-background silicon (911) substrate. The data were analysed using a Pawley fitting routine in the Topas Academic (version 6) software suite.

Single crystal X-ray diffraction

A suitable crystal of **1** with dimensions 0.48 × 0.33 × 0.22 mm^3 was selected and mounted on a MITIGEN holder in Paratone oil on a Rigaku Oxford Diffraction SuperNova diffractometer. The crystal was kept at a steady T = 120.00(10) K during data collection. The structure was solved using ShelXT[1] by dual methods and by using Olex2 1.5-beta[2] as the graphical interface. The model was refined with ShelXL[3] using full matrix least squares minimisation on F^2.

ESI-MS

ESI-MS measurements were recorded in positive and negative-ion mode using a Bruker ESI Micro-TOF spectrometer equipped with Liquid Chromatography (LC). All mass spectra were analyzed using Data Analysis software version 4.4 (Bruker Daltonics) with the ion peaks assigned manually.

UV-visible Spectrophotometry

The UV-vis experiments were analysed against a solvent blank over the range 200-1100 nm on a Shimadzu UV-1900 spectrometer.

NMR Spectroscopy

$^{31}P\{^1H\}$ NMR spectra were recorded on a Bruker Pro 500 spectrometer at 300 K at 202 MHz in CD_3CN. 85 % H_3PO_4 was used as an external standard at 0.00 ppm.

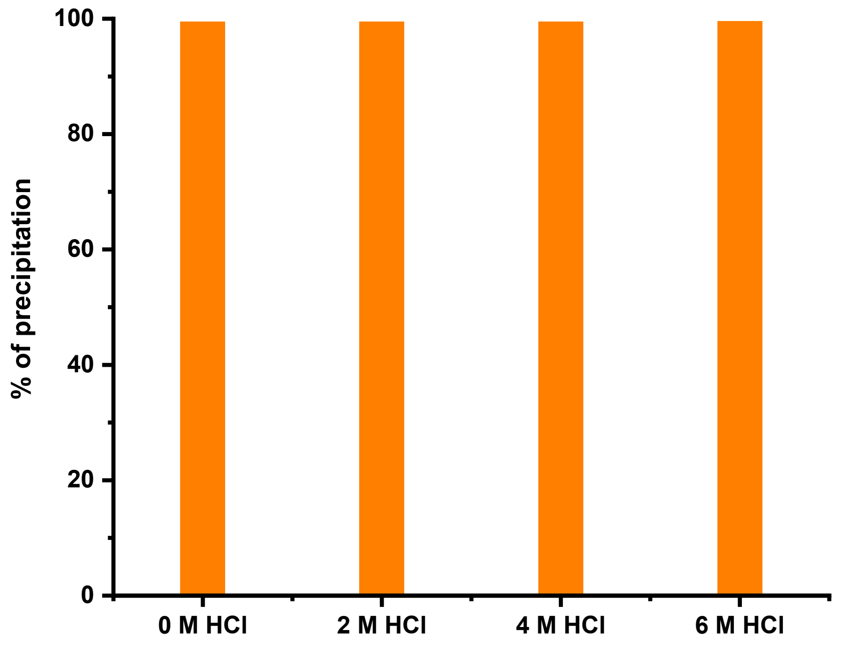

Figure S1. HCl concentration-dependent gold recovery from 3 mL of 10 mM $HAuCl_4$ solutions with 0.28 mmol of solid TPPO.

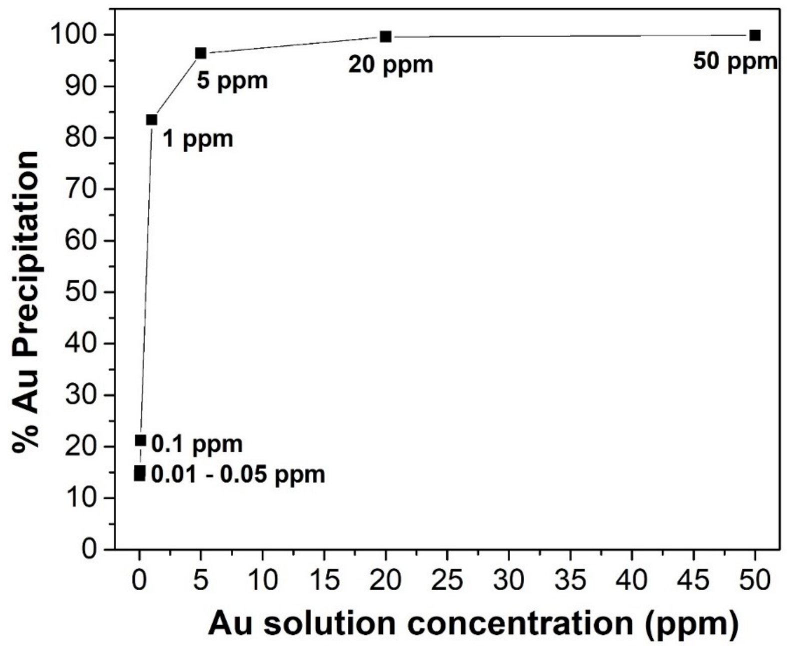

Figure S2. Graphical representation of percentage precipitation of gold from 0.01 ppm – 50.0 ppm gold solution in 2 M HCl using TPPO. Conditions: 5 mL $HAuCl_4$ solution contacted with 0.25 mmol of TPPO for 30 min. The solution was filtered, and 1 mL of each solution was taken out and further 10x diluted in 2% HNO_3 prior to ICP-MS analysis.

Table S1. Percentage precipitation of gold from 0.01 ppm – 50.0 ppm gold concentrated solution in 2 M HCl using TPPO. Conditions: 5 mL $HAuCl_4$ solution contacted with 0.25 mmol of TPPO for 30 min. The solution was filtered, and 1 mL of each solution was taken out and further 10x diluted in 2% HNO_3 prior to ICP-MS analysis.

Au Sample	Initial Au concentration (ppb)	Au concentration after contact with TPPO (ppb)	% Precipitated
0.01 ppm	0.778	0.679	12.7
0.05 ppm	3.976	3.288	17.3
0.1 ppm	8.328	6.267	24.7
1.0 ppm	86.802	14.502	83.3
5.0 ppm	442.219	15.613	96.5
20.0 ppm	1864.857	6.681	99.6
50.0 ppm	4511.612	3.603	99.9

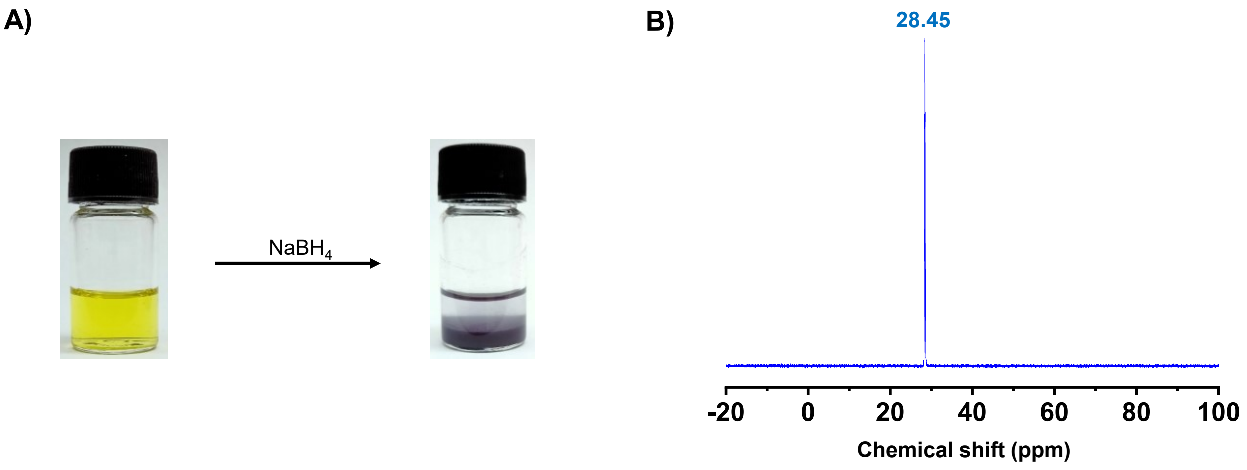

Figure S3. A) Reduction of $HAuCl_4$ to Au(0) using $NaBH_4$ (15 mg) in 3 mL acetonitrile to form a (black) Au precipitate (99%). **B)** $^{31}P\{^1H\}$ NMR spectrum of triphenylphosphine oxide that remains in acetonitrile after reduction.

Table S2. Crystal data and structure refinement for $[(TPPO)_4(H_5O_2)][AuCl_4]$ **1**

**Compound**	**JL22004**
CCDC number	2269430
Formula	$C_{72}H_{65}AuCl_4O_6P_4$
$D_{calc.}$/ g cm^{-3}	1.464
μ/mm^{-1}	2.482
Formula Weight	1488.88
Colour	translucent pale yellow
Shape	block-shaped
Size/mm^3	0.48×0.33×0.22
T/K	120.00(10)
Crystal System	triclinic
Space Group	P-1
a/Å	9.10360(10)
b/Å	13.7281(2)
c/Å	13.7355(2)
α/°	87.1290(10)
β/°	80.8740(10)
γ/°	85.6340(10)
V/Å^3	1688.69(4)
Z	1
Z'	0.5
Wavelength/Å	0.71073
Radiation type	Mo K_α
Θ_{min}/°	3.226
Θ_{max}/°	37.766
Measured Refl's.	83418
Indep't Refl's	17442
Refl's I≥2 σ(I)	16832
R_{int}	0.0463
Parameters	472
Restraints	15
Largest Peak	0.704
Deepest Hole	-1.475
GooF	1.049
wR_2 (all data)	0.0576
wR_2	0.0557
R_1 (all data)	0.0304
R_1	0.0276

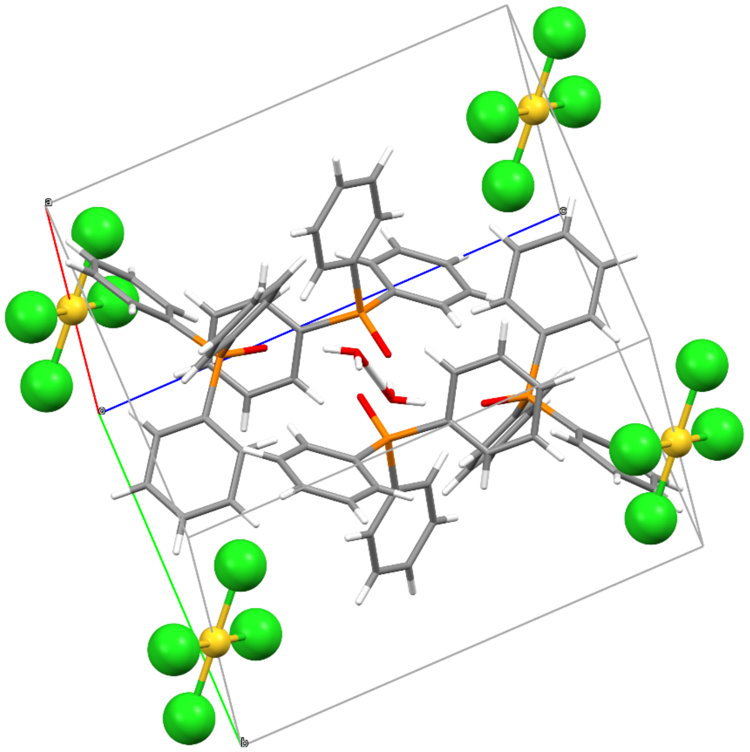

Figure S4. Unit cell diagram of [(TPPO)$_4$(H$_5$O$_2$)][AuCl$_4$] **1**.

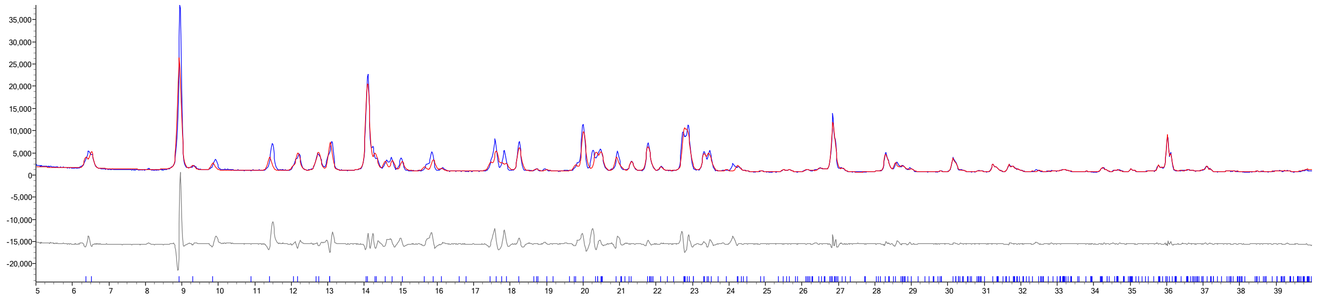

Figure S5. Powder X-ray diffraction of the precipitate showing the observed data (blue), the calculated profile (red) derived from the single-crystal X-ray structure of **1**, and the difference profile (grey). Blue tick marks are associated with [(TPPO)$_4$(H$_5$O$_2$)][AuCl$_4$] **1**.

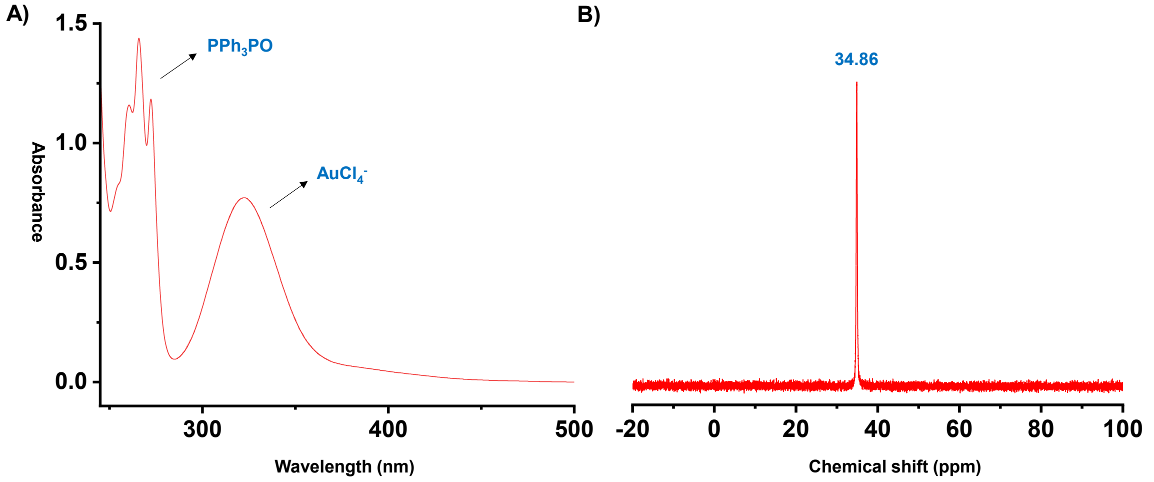

Figure S6. A) UV/Vis spectrum of **1** after dissolving it in acetonitrile consistent with the presence of $AuCl_4^-$ and Ph_3PO. **B)** $^{31}P\{^1H\}$ NMR spectrum of **1**. The peak at 40.1 ppm is assigned to the cation $(Ph_3PO)_2H^+$.

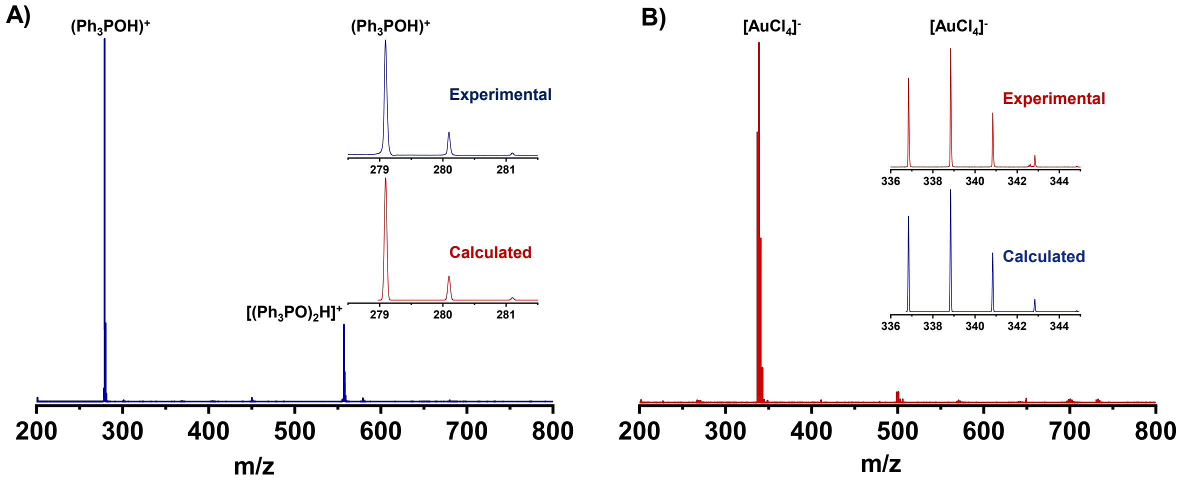

Figure S7. A) Positive-ion ESI-MS of **1** in acetonitrile showing the presence $(Ph_3PO)_2H^+$ and Ph_3POH^+. B) Negative ion ESI-MS of **1** in acetonitrile with observed and calculated isotopic distribution patterns for $AuCl_4^-$.

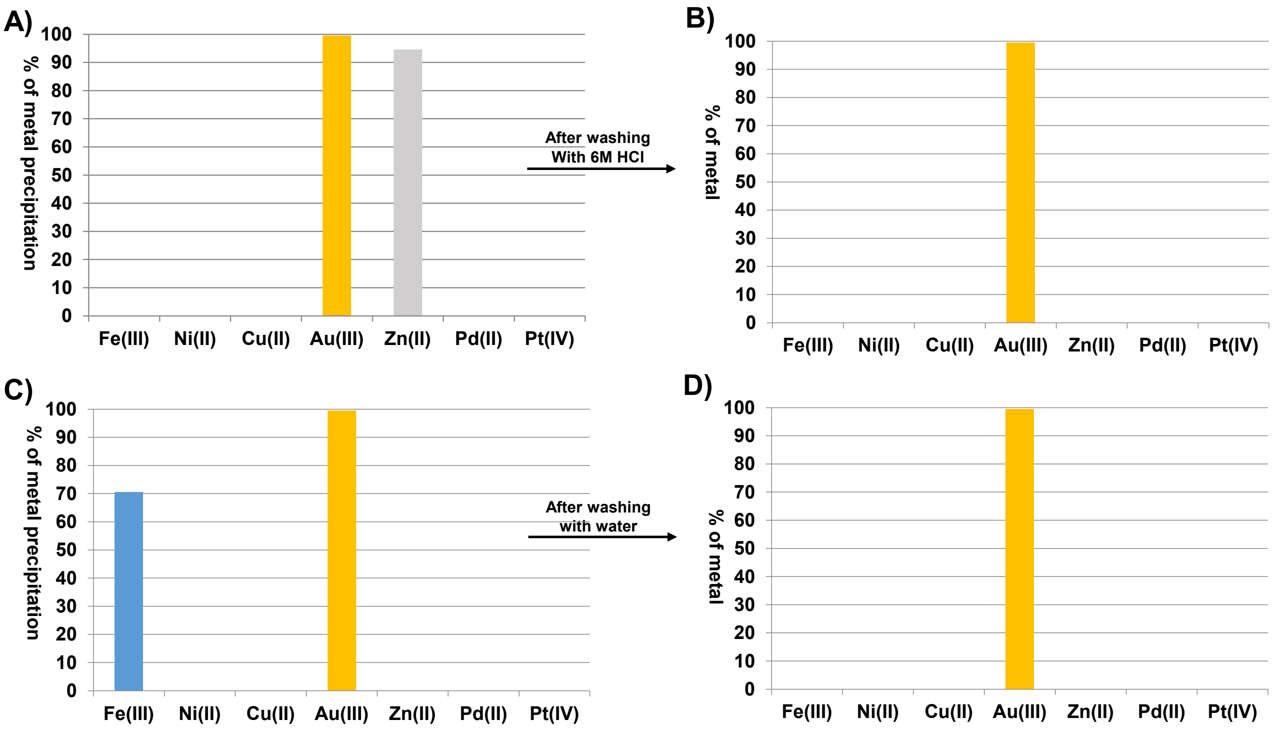

Figure S8. Selective recovery of metals from 10 mM mixed-metal solutions using TPPO. **A)** From 2 M HCl. **B)** After washing the precipitate obtained in A with 6 M HCl. **C)** From 6 M HCl. **D)** After washing the precipitate obtained in C with water.

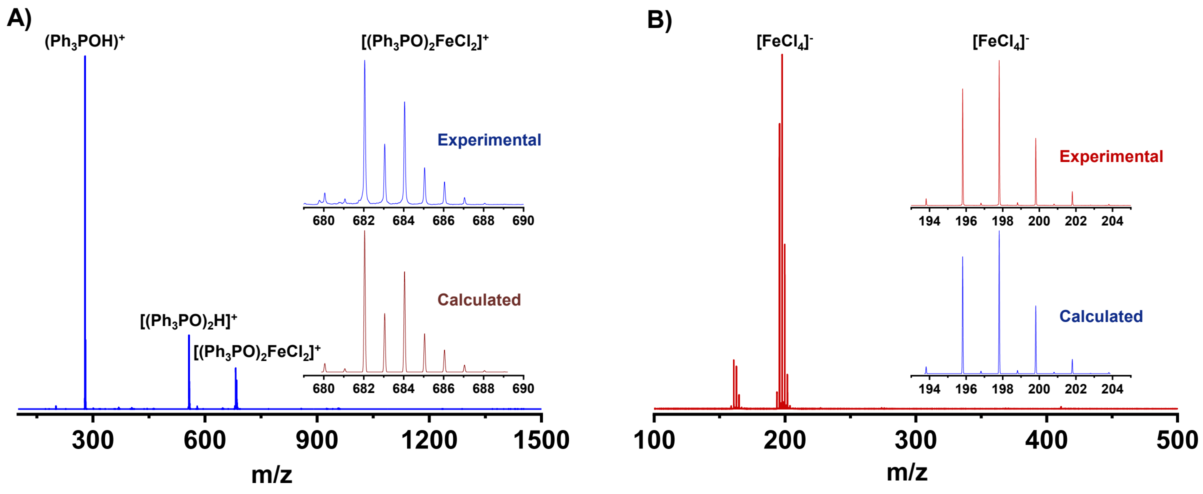

Figure S9. A) Positive-ion ESI-MS of $[FeCl_2(TPPO)_4][FeCl_4]$ after dissolving it in acetonitrile showing the presence $[FeCl_2(Ph_3PO)_2]^+$, $(Ph_3PO)_2H^+$, and Ph_3POH^+. B) Negative ion ESI-MS of the acetonitrile solution after dissolving $[FeCl_2(TPPO)_4][FeCl_4]$ with observed and calculated isotopic distribution patterns for $FeCl_4^-$.

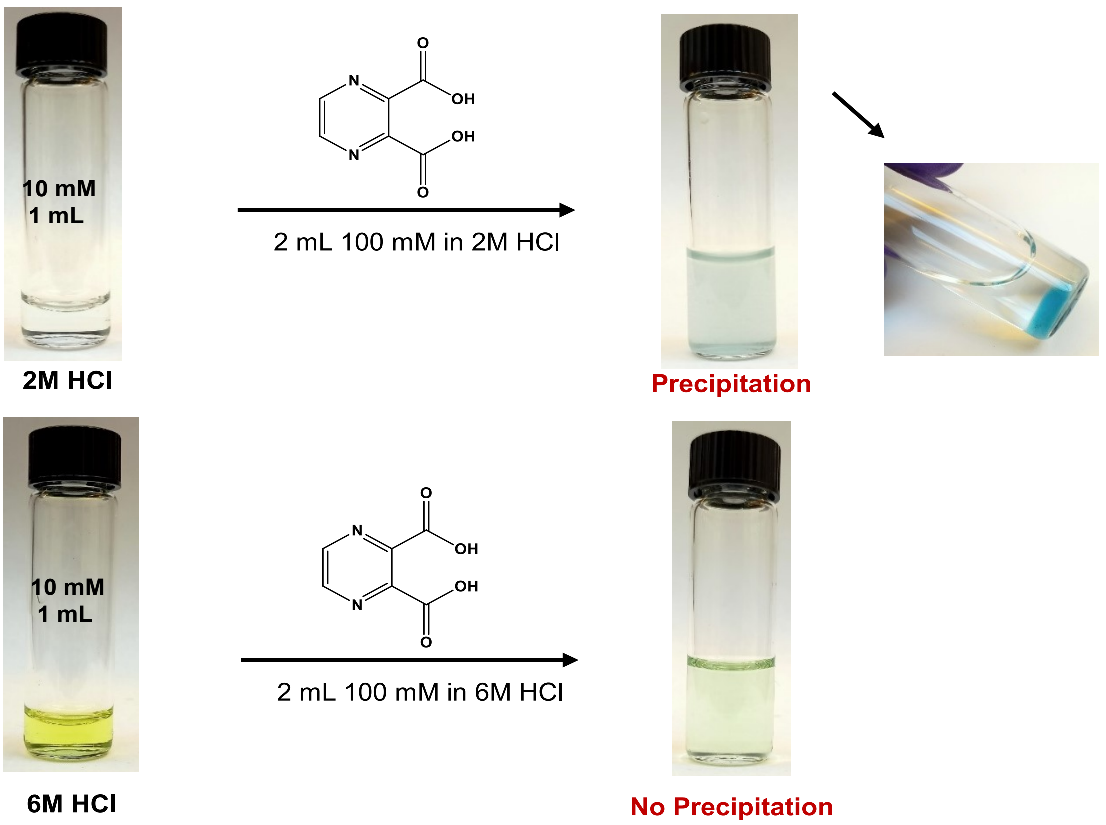

Figure S10. Complete precipitation (by ICP OES) of copper(II) chloride using 2,3-PDCA in 2 M HCl. No precipitation was observed from 6 M HCl.

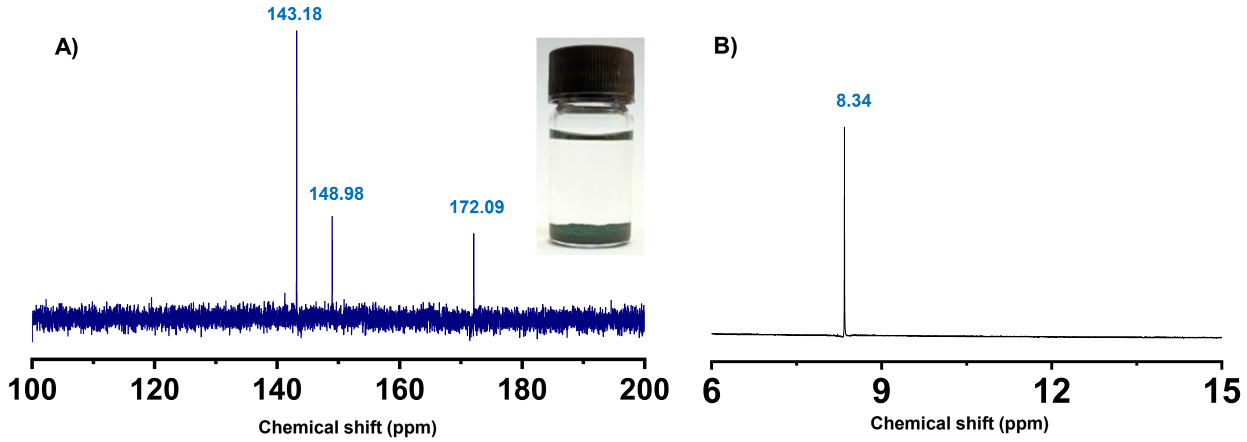

Figure S11: A) ^{13}C and B) ^{1}H NMR spectra (D_2O) of 2,3-PDCA that remained in aqueous medium after treatment with Na_2S. Photograph of the black CuS formed after treatment of the Cu PDCA complex with Na_2S is shown in the inset.

A) B)

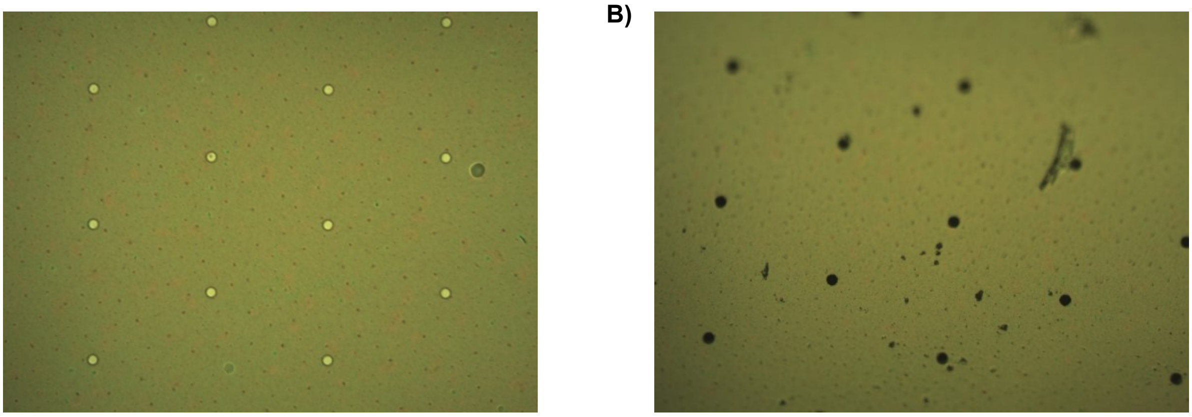

Figure S12: Photograph of platinum microelectrodes **A)** before and **B)** after the reduction of copper-2,3-PDCA complex in 6 M HCl by cyclic voltammetry. The black spots represent copper deposition to the electrode.

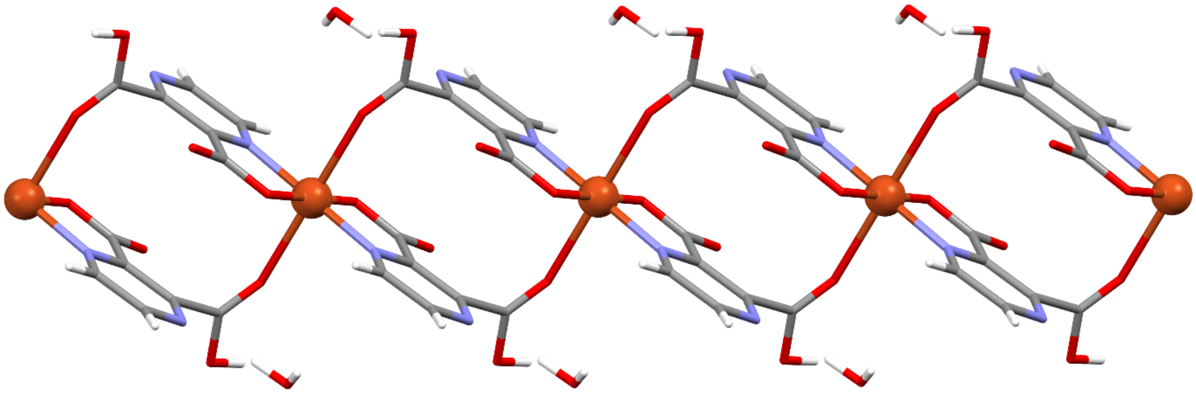

Figure S13: Extended structure of the copper-2,3-PDCA complex **2**.[4]

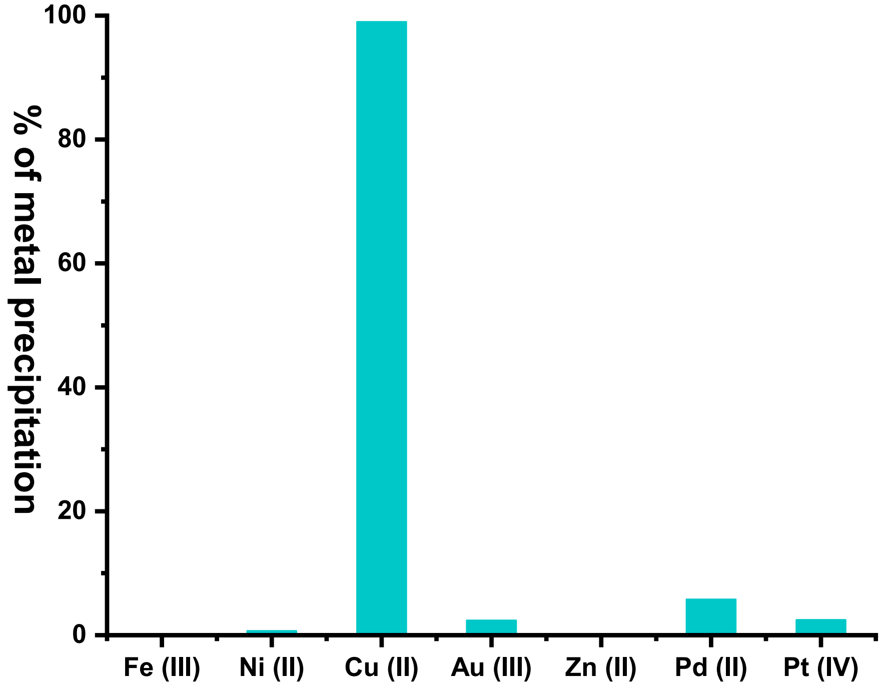

Figure S14: Percentages of metals recovered from a 10 mM mixed-metal solution in 2 M HCl using 2,3-PDCA.

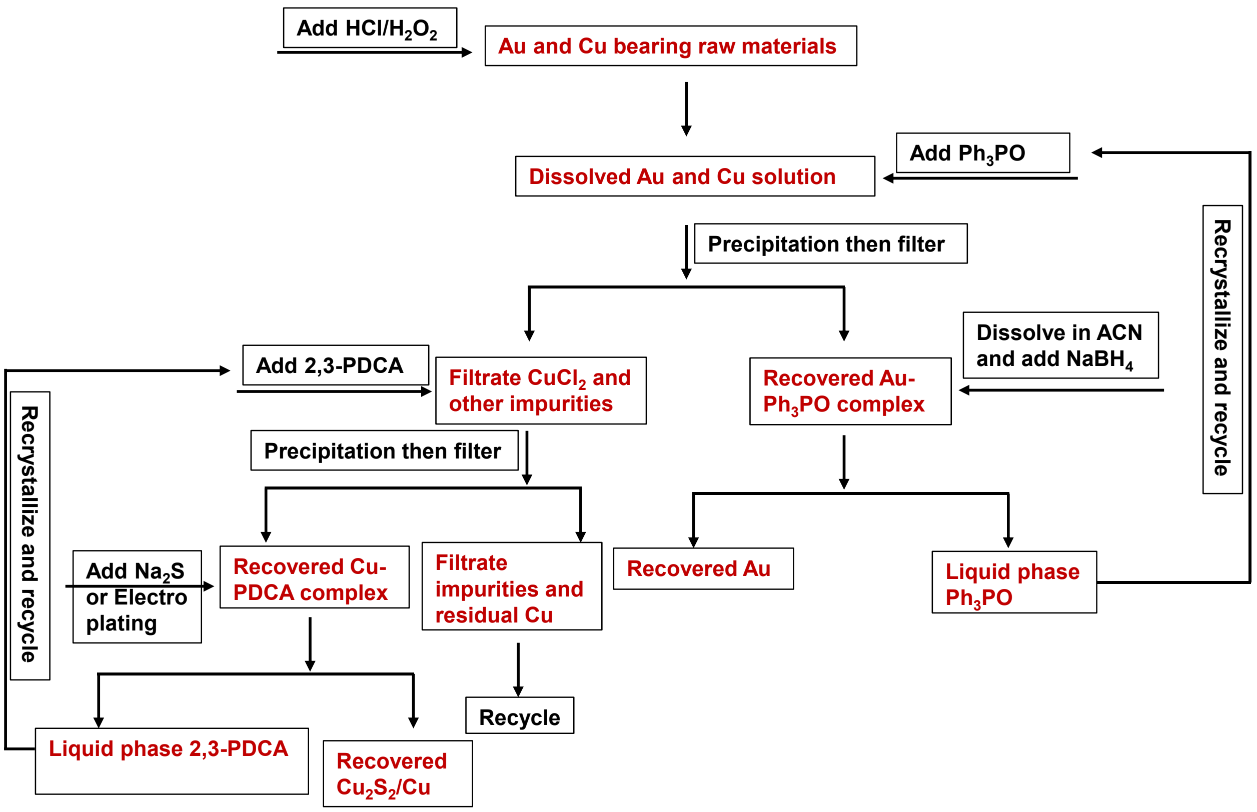

Figure S15: Gold and copper recovery flowchart.

References

[1] G. Sheldrick, *Acta Crystallogr. A* **2015**, *71*, 3-8.

[2] O. V. Dolomanov, L. J. Bourhis, R. J. Gildea, J. A. K. Howard, H. Puschmann, *J. Appl. Cryst.* **2009**, *42*, 339-341.

[3] G. Sheldrick, *Acta Crystallogr. C* **2015**, *71*, 3-8.

[4] L. Mao, S. J. Rettig, R. C. Thompson, J. Trotter, S. Xia, *Can. J. Chem.* **1996**, *74*, 433-444.

Author Contributions

Dr Abhijit Nag: investigation, methodology, data collection, data analysis, software, writing – original draft – review and editing; Dr Mukesh Kumar Singh: investigation, data collection, data analysis and writing – review and editing; Prof. Carole A. Morrison: supervision and writing – review and editing; Prof. Jason B. Love: data analysis, supervision and writing – review and editing.
